# Bacterial keratitis in patients with corneal lesions in southeastern Iran: microbial spectrum, demographic characteristics, and seasonal distribution

**DOI:** 10.1186/s40794-026-00322-2

**Published:** 2026-11-02

**Authors:** Mehdi Zarean, Ghodratollah Salehi Sangani, Mahsa Khodkari, Mojtaba Mousavi Bazaz, Soudabeh Etemadi

**Affiliations:** 1 Department of Parasitology and Mycology, Faculty of Medicine, Mashhad University of Medical Sciences, Mashhad, Iran; 2 Cutaneous Leishmaniosis Research Center, Mashhad University of Medical Sciences, Mashhad, Iran; 3 Student Research Committee, Faculty of Medicine, Mashhad University of Medical Sciences, Mashhad, Iran; 4 Department of Community Medicine, Faculty of Medicine, Mashhad University of Medical Sciences, Mashhad, Iran; 5 Department of Laboratory Sciences, Sirjan School of Medical Sciences, Sirjan, Iran

**Keywords:** Bacterial keratitis, Corneal ulcer, Epidemiology, *Staphylococcus epidermidis*, Southeastern Iran, Seasonal variation

## Abstract

**Background and objective:**

Bacterial keratitis is a sight-threatening ophthalmic emergency that can rapidly progress to corneal ulceration, perforation, endophthalmitis, and permanent blindness if inappropriately managed. Despite its clinical significance, there is a paucity of comprehensive regional microbiological surveillance data regarding the microbial etiology of bacterial keratitis in southeastern Iran. Therefore, this study aimed to provide an updated regional microbiological surveillance report, detailing the bacterial profile, seasonal distribution, and demographic patterns of bacterial keratitis in patients presenting with corneal lesions in this area.

**Materials and methods:**

This retrospective, descriptive cross-sectional study was conducted between 2020 and 2025. Data were retrieved from the medical registries of tertiary teaching hospitals affiliated with Zahedan University of Medical Sciences. Demographic variables (age, sex), seasonal onset, and microbiological isolates were extracted using standardized checklists. Patients with incomplete clinical or laboratory records were excluded. As a descriptive surveillance study, data were analyzed to establish local epidemiological baselines and pathogen distributions. Statistical analysis was performed using SPSS version 26.

**Results:**

A total of 358 patients were evaluated, comprising 217 males (60.6%) and 141 females (39.4%). While bacterial isolates showed numerical variations across seasons (higher in Spring/Summer: 52.0% vs. Autumn/Winter: 48.0%) and genders (males: 60.6%), these demographic and seasonal distributions were not statistically significant (p > 0.05p > 0.05). The five most prevalent bacterial isolates were Staphylococcus epidermidis (n = 126, 35.2%), Staphylococcus saprophyticus (n = 59, 16.5%), Streptococcus pneumoniae (n = 50, 14.0%), Pseudomonas spp. (n = 37, 10.3%), and Acinetobacter spp. (n = 23, 6.4%). Regarding age distribution, S. epidermidis was most frequent in patients aged ≤ 20 years (n = 40) and ≥ 61 years (n = 37), whereas S. saprophyticus peaked in the ≤ 20 years age group (n = 27). Furthermore, Streptococcus pneumoniae (n = 23), Pseudomonas spp. (n = 15), and Acinetobacter spp. (n = 10) showed higher frequencies in older adults aged ≥ 61 years. Temporally, peak numerical frequencies of S. epidermidis, S. saprophyticus, and Acinetobacter spp. occurred in summer, while S. pneumoniae and Pseudomonas spp. peaked in autumn. All major isolates exhibited a higher numerical frequency in male patients.

**Conclusion:**

Bacterial keratitis in southeastern Iran is characterized by a predominance of Gram-positive cocci, with Staphylococcus epidermidis being the most common isolate. Although numerical variations across seasons and genders were observed, they lacked statistical significance. Consequently, empirical therapy and clinical management should remain consistent throughout the year. Based on the observed microbial spectrum, broad-spectrum empirical therapy targeting both Gram-positive and Gram-negative pathogens should be initiated promptly to optimize clinical outcomes and mitigate visual loss.

**Graphical Abstract:**

**Figure:**
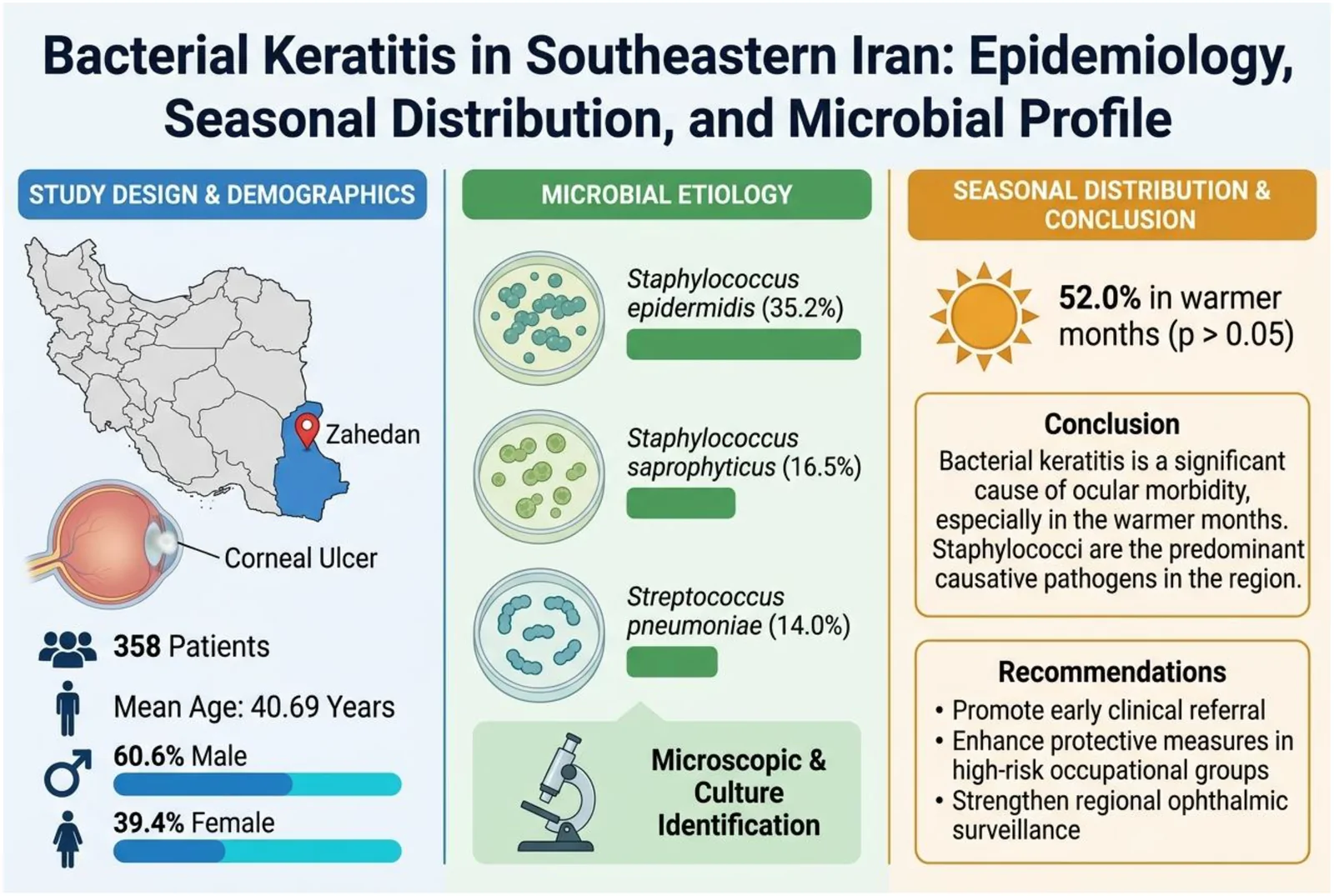

## Introduction

Infectious keratitis, characterized by corneal ulceration and subsequent opacity, represents a critical global cause of visual impairment. It is etiologically classified into microbial (bacterial, fungal, or parasitic) and viral (typically herpes simplex) subtypes [1, 2]. Among these, bacterial keratitis (BK) is the most prevalent form and is clinically managed as an ophthalmic emergency due to its potential for rapid progression [3, 4]. The clinical severity of BK is stratified based on ulcer size and stromal depth: mild cases involve lesions < 2 mm with < 20% stromal thickness involvement; moderate cases present with 2–5 mm lesions and 20–50% depth (100–275 $\:\mu\:m$); while severe keratitis is defined by lesions $\:\ge\:$5 mm or depth exceeding 50% (> 275 $\:\mu\:m$), posing a substantial risk for irreversible visual loss [5, 6].

The incidence and microbiological spectrum of BK exhibit significant geographical variation, influenced by local climate and environmental risk factors [7]. A distinct disparity exists between developed and developing regions; for instance, while the United States reports approximately 71,000 cases annually—driven largely by contact lens wear—developing nations like India and Pakistan face different challenges [2, 8–10]. While *Staphylococcus* spp. and *Pseudomonas aeruginosa* are the most frequently reported pathogens in Western cohorts, studies in North America and Europe highlight a shift towards Gram-negative organisms in southern latitudes [11–17]. Furthermore, polymicrobial infections, often involving *Staphylococcus epidermidis* and *Fusarium* spp. following ocular trauma, complicate the clinical landscape and necessitate precise microbiological identification [18, 19].

Despite advancements in molecular diagnostics and targeted antimicrobial therapies, BK remains a leading cause of blindness in underdeveloped and rural areas [7]. The microbial profile of keratitis is dynamic, with recent decades showing a surge in incidence linked to changing environmental conditions and risk factors. In Iran, while some data exist for other regions, there is a notable paucity of epidemiological research regarding the specific bacterial agents and seasonal distribution of keratitis in the southeastern part of the country. Consequently, this study was designed to investigate the bacterial etiology and demographic patterns of corneal lesions in southeastern Iran, providing essential data to optimize regional empirical treatment protocols and improve visual outcomes.

## Materials and methods

### Study design and setting

**Fig. 1:**
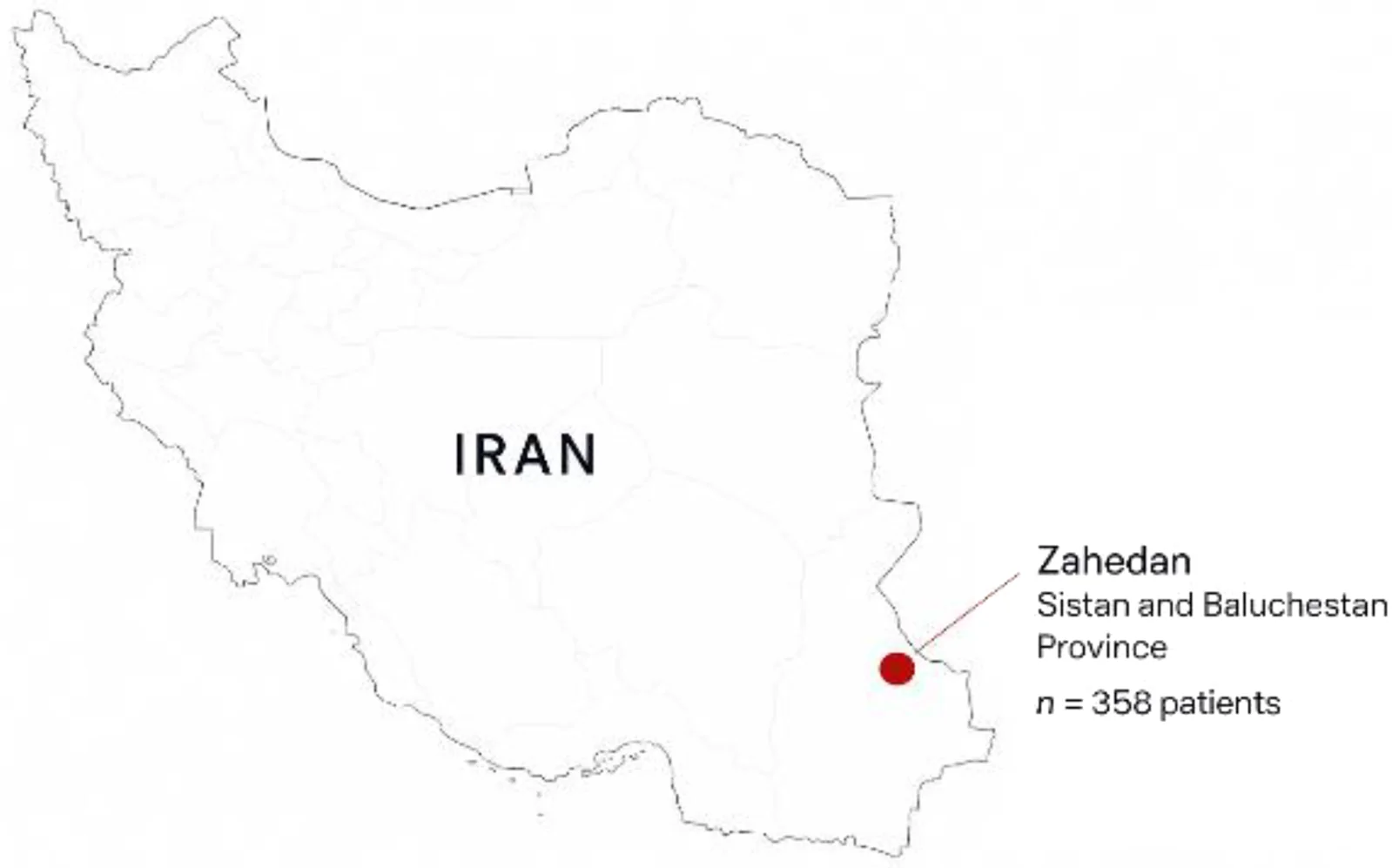
Geographic location of the study site in Zahedan, Sistan and Baluchestan Province, Iran. This study was conducted on a cohort of 358 patients with clinically diagnosed bacterial keratitis

This retrospective, descriptive, cross-sectional study was conducted to investigate the bacterial etiology of corneal lesions among patients referred to tertiary teaching hospitals affiliated with Zahedan University of Medical Sciences, southeastern Iran. The study period spanned five years, from 1399–1403(2020 to 2025). The geographic location of the study site is illustrated in Fig. 1.

### Study population and sampling

A census sampling approach was employed, encompassing all patients who presented with clinical signs of infectious keratitis during the study period. The inclusion criteria comprised a definitive clinical diagnosis of bacterial keratitis—characterized by observable corneal infiltration and conjunctival injection—along with the availability of comprehensive medical and microbiological records. Bacterial isolates recovered from corneal scrapings were identified using standard laboratory protocols. Gram-positive, catalase-positive cocci were further evaluated using the tube coagulase test. Species-level identification of coagulase-negative staphylococci (CoNS), including *Staphylococcus epidermidis and Staphylococcus* saprophyticus, was performed using standardized conventional biochemical and enzymatic profiling panels (such as carbohydrate fermentation and urease tests) as per standard laboratory procedures.

To minimize the confounding effect of potential ocular surface contaminants (such as resident skin flora), strict criteria were applied: coagulase-negative staphylococci (*Staphylococcus epidermidis* and *Staphylococcus saprophyticus*) were designated as true pathogens only after a rigorous retrospective review of medical and microbiological charts. Specifically, their clinical significance required documented evidence of localized corneal inflammation (e.g., stromal infiltrates with suppuration) combined with supportive laboratory findings, such as heavy or confluent growth on primary culture media or consistent observations of Gram-positive cocci on direct Gram-stained smears. Conversely, patients with incomplete demographic data, missing laboratory results, or inaccessible medical records were excluded. Ultimately, a total of 358 patients met all inclusion criteria and were enrolled in the study.

### Data collection and variables

Data were systematically extracted from the hospital’s integrated health information systems and laboratory registries using standardized checklists. The collected variables included demographic characteristics (age and sex), clinical history, date of presentation, seasonal timing of infection, and the specific bacterial isolates identified through microbiological culture and identification methods. To ensure data integrity, all information was double-checked by two independent researchers before being entered into the final database.

#### Antimicrobial Susceptibility Testing (AST)

The antimicrobial susceptibility of the bacterial isolates was determined using the Kirby-Bauer disk diffusion method. All testing and interpretation were performed in strict accordance with the Clinical and Laboratory Standards Institute (CLSI) guidelines, specifically the M100 version 33 (2023). Organism-specific susceptibility breakpoints were selected based on the standardized tables provided in the aforementioned CLSI manual. To ensure the accuracy and reliability of the susceptibility results, quality control procedures were implemented using standard reference control strains, following the established CLSI protocols.

Antimicrobial susceptibility testing (AST) was performed on a clinical subset of the isolated organisms as part of routine diagnostic microbiology workflows, depending on sample viability, secondary subculturing success, and clinical requirements. Consequently, susceptibility data were not uniformly available for every confirmed isolate, resulting in varying denominators across the evaluation panels.

### Statistical analysis

Statistical analysis was performed using IBM SPSS Statistics for Windows, version 26 (IBM Corp., Armonk, NY, USA). The normality of quantitative data distribution was assessed using the Kolmogorov-Smirnov test. Quantitative variables were expressed as median and interquartile range (IQR), while qualitative variables were presented as absolute frequencies and percentages. To account for sparse categorical data and small expected cell frequencies across contingency tables, the Fisher-Freeman-Halton exact test was applied. A *p-value* of less than 0.05 was considered statistically significant for all tests.

## Results

**Fig. 2:**
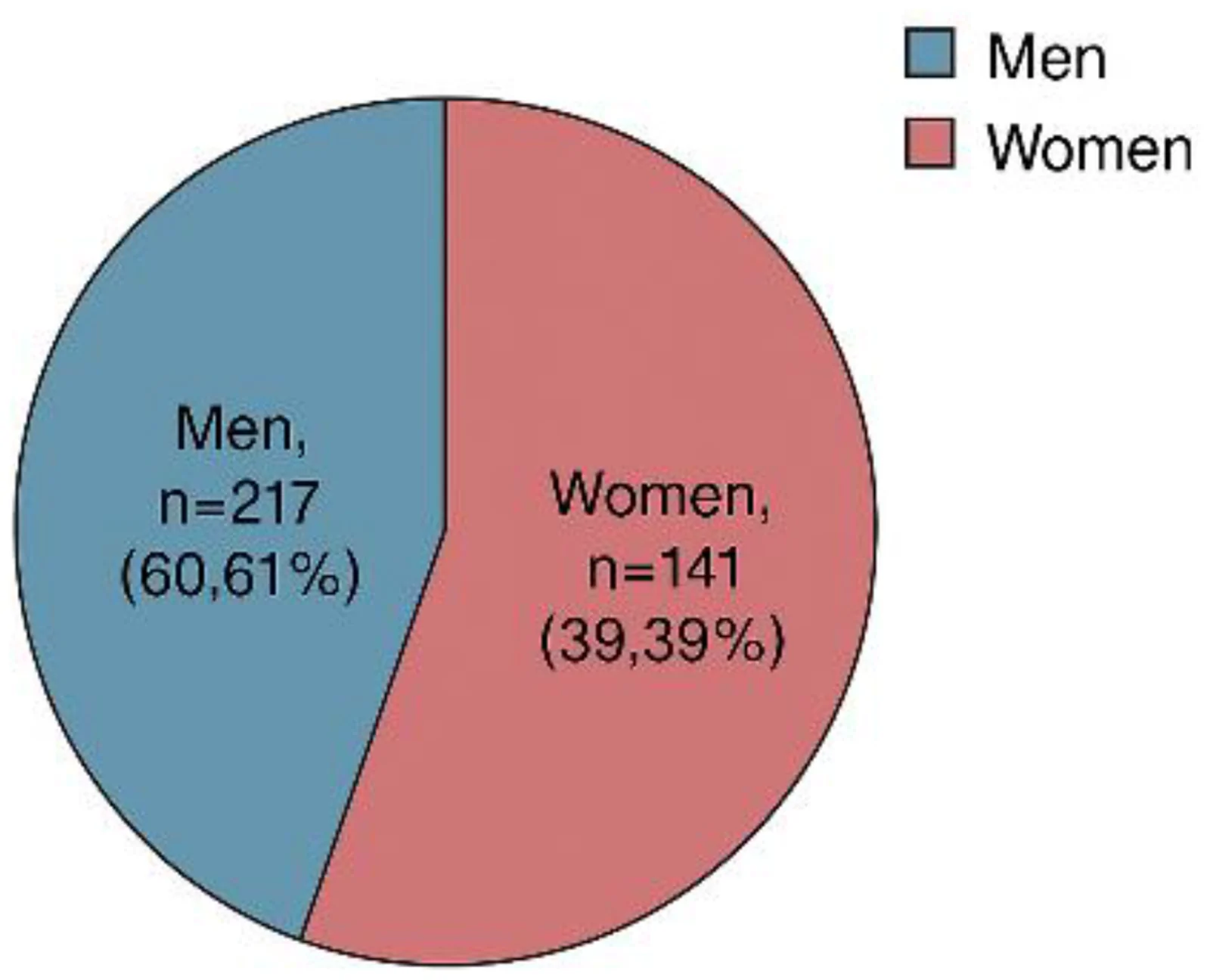
Demographic distribution of patients with bacterial keratitis by age and sex in southeastern Iran. (*n* = 358)

**Fig. 3:**
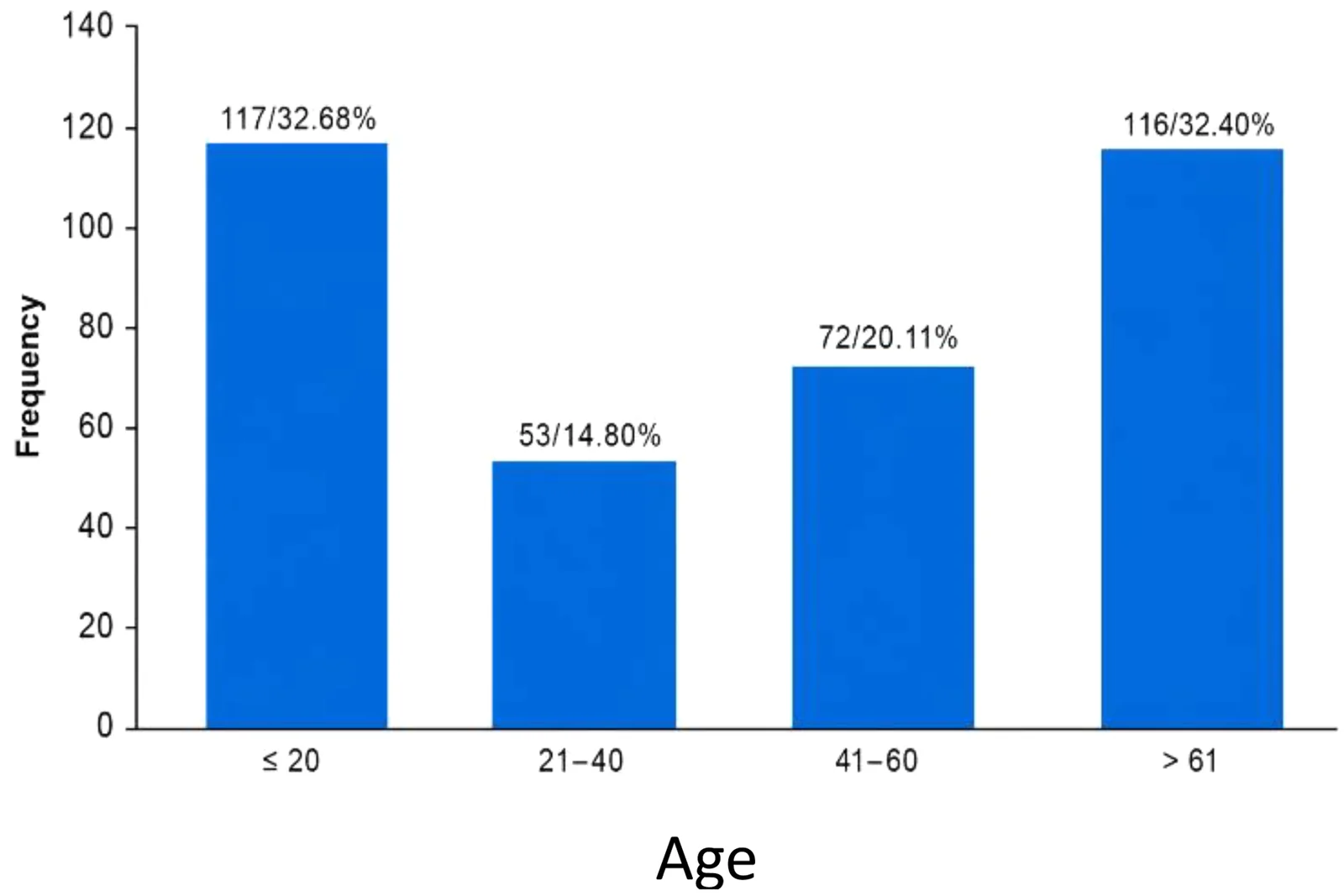
Demographic distribution of patients with bacterial keratitis by age and sex in southeastern Iran. (*n* = 358)

During the study period, a total of 358 patients who met the inclusion criteria were evaluated (Figs. 2 and 3).

The five most frequently identified isolates in patients with bacterial keratitis were, in descending order, *Staphylococcus epidermidis* (126 cases, 35.2%), *Staphylococcus saprophyticus* (59 cases, 16.5%), *Streptococcus pneumoniae* (50 cases, 14.0%), *Pseudomonas* spp. (37 cases, 10.3%) *Acinetobacter* spp. (23 cases, 6.4%), and. (Table 1).

**Table 1:** Frequency and distribution of bacterial isolates in patients with bacterial keratitis (*N* = 358)

Microorganism	Frequency (*n*)	Percentage (%)
Staphylococcus epidermidis	126	35.2
Staphylococcus saprophyticus	59	16.5
Streptococcus pneumoniae	50	14.0
Pseudomonas spp.	37	10.3
Acinetobacter spp.	23	6.4
Staphylococcus aureus	17	4.7
Klebsiella spp.	16	4.5
Escherichia coli	8	2.2
Enterococcus spp.	8	2.2
Streptococcus viridans	5	1.4
Haemophilus spp.	3	0.8
Other Staphylococci	3	0.8
Enterobacter spp.	2	0.6
Corynebacterium diphtheriae	1	0.3
Total	358	100.0

The distribution of bacterial isolates across age groups is shown in Table 2. *Staphylococcus epidermidis* and *Staphylococcus saprophyticus*** were** more prevalent in the youngest group ($\:\le\:20$ years, * n=40*and * 27*, respectively), while *Streptococcus pneumoniae*, *Pseudomonas* spp., and *Acinetobacter* spp. showed higher frequencies in patients aged $\:\ge\:61$years (* n=23*, * 15*, and * 10*, respectively). There is no statistically significant difference in the frequency distribution of bacterial isolates across different age groups.) *P*=0.748).

**Table 2:** Age-based distribution of bacterial isolates causing bacterial keratitis

Causative organism	≤ 20 years*	21–40 years	41–60 years	≥ 61 years	Total
Staphylococcus epidermidis	40	26	23	37	126
Staphylococcus saprophyticus	27	7	12	13	59
Streptococcus pneumoniae	10	5	12	23	50
Pseudomonas spp.	7	6	9	15	37
Acinetobacter spp.	7	4	2	10	23
Staphylococcus aureus	7	3	4	3	17
Klebsiella spp.	8	0	3	5	16
Escherichia coli	2	1	2	3	8
Enterococcus spp.	3	1	3	1	8
Streptococcus viridans	2	0	0	3	5
Haemophilus spp.	2	0	0	1	3
Other Staphylococcus spp.	2	0	1	0	3
Enterobacter spp.	0	0	1	1	2
Corynebacterium diphtheriae	0	0	0	1	1
Total	117	53	72	116	358

The seasonal distribution of bacterial isolates is presented in Table 3. Staphylococcus epidermidis was consistently the most prevalent organism across all seasons: spring (*n* = 35), summer (*n* = 37), autumn (*n* = 29), and winter (*n* = 25). Regarding the second most frequent isolate, Staphylococcus saprophyticus predominated in spring, summer, and winter; however, Streptococcus pneumoniae emerged as the second most common isolate during the autumn season. Notably, the highest volume of keratitis cases was recorded during the summer (*n* = 103). The seasonal distribution of bacterial isolates causing keratitis did not show any statistically significant difference (*p = 0.474*).

**Table 3:** Seasonal distribution of bacterial isolates causing keratitis and comparison across seasons

Causative organism	Spring	Summer	Autumn	Winter	Total
Staphylococcus epidermidis	35	37	29	25	126
Staphylococcus saprophyticus	11	21	13	14	59
Streptococcus pneumoniae	9	14	15	12	50
Pseudomonas spp.	7	6	13	11	37
Acinetobacter spp.	4	9	6	4	23
Staphylococcus aureus	3	4	5	5	17
Klebsiella spp.	5	4	1	6	16
Escherichia coli	3	1	1	3	8
Enterococcus spp.	1	1	5	1	8
Streptococcus viridans	2	1	2	0	5
Haemophilus spp.	1	1	1	0	3
Other Staphylococcus spp.	1	2	0	0	3
Enterobacter spp.	0	2	0	0	2
Corynebacterium diphtheriae	1	0	0	0	1
Total	83	103	91	81	358

**Table 4:** Frequency and distribution of isolated bacterial pathogens by gender (*N* = 358)

Bacterial Isolates	Male (*n* = 217)	Female (*n* = 141)	Total (*N* = 358)	Percentage (%)
Staphylococcus epidermidis	80	46	126	35.2%
Staphylococcus saprophyticus	34	25	59	16.5%
Streptococcus pneumoniae	33	17	50	14.0%
Pseudomonas spp	26	11	37	10.3
Acinetobacter spp.	15	8	23	6.4%
Staphylococcus aureus	10	7	17	4.7%
Klebsiella spp.	7	9	16	4.5%
Escherichia coli	3	5	8	2.2%
Enterococcus spp.	2	6	8	2.2%
Streptococcus viridans	2	3	5	1.4%
Haemophilus spp.	2	1	3	0.8%
Staphylococcus spp.	2	1	3	0.8%
Enterobacter spp.	0	2	2	0.6%
Corynebacterium diphtheriae	1	0	1	0.3%
Total	217	141	358	100%

The distribution of bacterial isolates by gender is presented in Table 4. The distribution of bacterial isolates was analyzed by gender using the Fisher-Freeman-Halton exact test (due to small cell counts). There was no statistically significant association between gender and the type of bacterial pathogens isolated (*P* = 0.562).

The organism frequencies reported in the descriptive analysis represent all eligible culture-positive isolates identified during the study period. However, it should be noted that antimicrobial susceptibility testing was not consistently available for every isolate across all antibiotic panels. Consequently, the denominators in Tables 5, 6, 7 and 8 vary by organism and antibiotic, reflecting missing or non-interpretable results in the retrospective laboratory records.

### In vitro susceptibility of bacterial keratitis causative agents to antibiotics

The antibiotic susceptibility profiles for Pseudomonas isolates (*N* = 17) are summarized in Table 5. Among the tested agents with established CLSI clinical breakpoints, the isolates exhibited the highest susceptibility to ciprofloxacin (76.5%, 13/17), followed by gentamicin (70.6%, 12/17), tobramycin (70.6%, 12/17), and ceftazidime (58.8%, 10/17). These findings underscore that fluoroquinolones and aminoglycosides remain the most effective therapeutic options for the management of Pseudomonas-associated corneal infections in this study population. Antibiotics with intrinsic resistance or lacking relevant interpretive criteria for this genus were excluded from the analysis to ensure clinical validity.

**Table 5:** Antibiotic susceptibility profiles of *Pseudomonas* isolates (*N* = 17)

Antibiotic	Resistant *n* (%)	Intermediate *n* (%)	Susceptible *n* (%)	Total (*N*)
Ceftazidime	4 (23.5)	3 (17.6)	10 (58.8)	17
Ciprofloxacin	4 (23.5)	0 (0.0)	13 (76.5)	17
Gentamicin	5 (29.4)	0 (0.0)	12 (70.6)	17
Tobramycin	3 (17.6)	2 (11.8)	12 (70.6)	17

The antibiotic susceptibility profiles for *Staphylococcus epidermidis* are summarized in Table 6. Among the tested antibiotics, cefazolin demonstrated the highest efficacy, with a susceptibility rate of 84.9% (*n* = 101/119), followed by cephalothin (83.2%, *n* = 99/119) and tobramycin (81.1%, *n* = 99/122). Resistance was most frequently observed against ciprofloxacin, affecting 19.5% (*n* = 22/113) of the isolates. Overall, these findings suggest that first-generation cephalosporins remain effective treatment options for *S. epidermidis* in this study population, exhibiting superior susceptibility profiles compared to the tested fluoroquinolones.

**Table 6:** Antibiotic susceptibility profiles of *Staphylococcus epidermidis* isolates

Antibiotic	Resistant *n* (%)	Intermediate *n* (%)	Susceptible *n* (%)	Total (*N*)
Cefazolin	7 (5.9)	11 (9.2)	101 (84.9)	119
Cephalothin	7 (5.9)	13 (10.9)	99 (83.2)	119
Tobramycin	9 (7.4)	14 (11.5)	99 (81.1)	122
Ciprofloxacin	22 (19.5)	19 (16.8)	72 (63.7)	113

The antibiotic susceptibility profiles for *Staphylococcus aureus* are presented in Table 7. Tobramycin demonstrated 100% susceptibility (*n* = 16/16), followed by cephalothin at 87.5% (*n* = 14/16). Regarding cefazolin, 43.8% (*n* = 7/16) of the isolates were resistant, while 56.3% (*n* = 9/16) were susceptible. For tetracycline, 43.8% (*n* = 7/16) of the isolates were resistant, while 31.3% (*n* = 5/16) showed intermediate resistance. Overall, these findings highlight a high susceptibility to aminoglycosides and first-generation cephalosporins in our study population, alongside notable resistance to tetracycline.

**Table 7:** Antibiotic susceptibility profiles of *Staphylococcus aureus* isolates

Antibiotic	Resistant *n* (%)	Intermediate *n* (%)	Susceptible *n* (%)	Total (*N*)
Tobramycin	0 (0.0)	0 (0.0)	16 (100.0)	16
Cephalothin	2 (12.5)	0 (0.0)	14 (87.5)	16
Cefazolin	7 (43.8)	0 (0.0)	9 (56.3)	16
Tetracycline	7 (43.8)	5 (31.3)	4 (25.0)	16

The antibiotic susceptibility profiles for *Streptococcus pneumoniae* are summarized in Table 8. The isolates demonstrated high susceptibility rates to vancomycin (91.5%, *n* = 43/47), cephalothin (91.3%, *n* = 42/46), cefazolin (89.6%, *n* = 43/48), and ciprofloxacin (80.9%, *n* = 38/47), with no resistant strains detected for vancomycin or ciprofloxacin.

**Table 8:** Antibiotic susceptibility profiles of *Streptococcus pneumoniae* isolates

Antibiotic	Resistant *n* (%)	Intermediate *n* (%)	Susceptible *n* (%)	Total (*N*)
Cephalothin	2 (4.3)	2 (4.3)	42 (91.3)	46
Vancomycin	0 (0.0)	4 (8.5)	43 (91.5)	47
Ciprofloxacin	0 (0.0)	9 (19.1)	38 (80.9)	47
Cefazolin	3 (6.3)	2 (4.2)	43 (89.6)	48

The antibiotic susceptibility profiles for Acinetobacter isolates are summarized in Table 9. Ciprofloxacin demonstrated the highest susceptibility rate at 69.6% (*n* = 16/23), followed by gentamicin at 63.6% (*n* = 14/22). Ceftriaxone showed 47.6% (*n* = 10/21) susceptibility, with a 33.3% resistance rate.

**Table 9:** Antibiotic susceptibility profiles of ***Acinetobacter*** isolates

Antibiotic	Resistant *n* (%)	Intermediate *n* (%)	Susceptible *n* (%)	Total (*N*)
Ciprofloxacin	6 (26.1)	1 (4.3)	16 (69.6)	23
Ceftriaxone	7 (33.3)	4 (19.0)	10 (47.6)	21
Gentamicin	4 (18.2)	4 (18.2)	14 (63.6)	22

The antibiotic susceptibility profiles for *Staphylococcus saprophyticus* isolates are detailed in Table 10. Among the tested agents, ciprofloxacin demonstrated the most favorable susceptibility profile, with 69.6% (*n* = 39/56) of isolates categorized as susceptible. Conversely, the highest resistance rate was observed against cephalothin at 17.0% (*n* = 9/53). Tobramycin and cefazolin also showed moderate activity, with susceptibility rates of 65.5% (*n* = 38/58) and 61.4% (*n* = 35/57), respectively.

**Table 10:** Antibiotic susceptibility profiles of *Staphylococcus saprophyticus* isolates

Antibiotic	Resistant *n* (%)	Intermediate *n* (%)	Susceptible *n* (%)	Total (*N*)
Cephalothin	9 (17.0)	11 (20.8)	33 (62.3)	53
Ciprofloxacin	9 (16.1)	8 (14.3)	39 (69.6)	56
Tobramycin	7 (12.1)	13 (22.4)	38 (65.5)	58
Cefazolin	9 (15.8)	13 (22.8)	35 (61.4)	57

## Discussion

The microbial spectrum observed in our study exhibits distinct variations when compared to other regional studies within Iran, reinforcing the value of local microbiological surveillance. For instance, in a study by Soleimani et al. (2022) conducted in Tehran, *Pseudomonas aeruginosa* was identified as the predominant pathogen in younger patients, whereas *Streptococcus pneumoniae* was more prevalent in patients over 50 years of age [20]. Similarly, Rahimi et al. (2015) in central Iran reported *P. aeruginosa* and *S. pneumoniae* as the most common isolates [21]. In contrast, Oryan, Faghri et al. (2014) reported a higher prevalence of coagulase-negative staphylococci (*Staphylococcus epidermidis*) in their study in Isfahan [22]. These disparities underscore the heterogeneous nature of the microbial landscape and the necessity of province-specific surveillance data across Iran [22].

Our findings revealed that the highest rate of patient admissions occurred in 2019 (* n=130*, $\:36.3\%$), while the lowest was recorded in 2022 ($\:1.7\%$). The five most prevalent pathogens identified were *Staphylococcus epidermidis* ($\:35.2\%$), followed by *Staphylococcus saprophyticus* ($\:16.5\%$), *Streptococcus pneumoniae* ($\:14.0\%$), *Acinetobacter* spp. ($\:6.4\%$), and *Pseudomonas* spp. ($\:10.3\%$). Consistent with our findings, Tavassoli et al. (2019) evaluated 2,116 patients with corneal abrasions and reported that coagulase-negative staphylococci (CoNS) were the most frequently cultured organisms (* n=430*), with *Pseudomonas aeruginosa* representing the most common Gram-negative isolate (* n=189*) [23]. Similarly, Peng et al. (2018) analyzed 2,203 corneal cultures, obtaining positive bacterial growth in $\:23.7\%$(* n=522*) of cases; of these, Gram-positive pathogens dominated ($\:65.3\%$, * n=338*), with methicillin-sensitive *Staphylococcus aureus* (MSSA) being the most prevalent ($\:20.1\%$, * n=105*), while Gram-negative bacilli accounted for $\:34.7\%$(* n=180*), led by *P. aeruginosa* ($\:10.9\%$, * n=57*) [24]. Furthermore, Tam et al. (2017) confirmed that CoNS and *P. aeruginosa* remained the leading Gram-positive and Gram-negative bacterial keratitis pathogens, respectively, over a 16-year surveillance period in Toronto [25].

Our surveillance data revealed distinct demographic patterns in microbial profiles; however, while descriptive variations were noted—such as the predominance of Gram-positive isolates like S. epidermidis and S. saprophyticus in patients under 20 years, and a higher frequency of S. pneumoniae, Acinetobacter, and Pseudomonas in those older than 61—these age-related trends require cautious interpretation within the context of local microbiological monitoring. Furthermore, all five leading isolates showed a numerical male predominance, which might reflect potential differences in healthcare-seeking behaviors or baseline population characteristics within this region; nevertheless, these factors remain speculative and warrant further targeted analytical investigation.

Regarding environmental and climatic context, southeastern Iran experiences frequent dust and sandstorms. While such arid-environment exposures are notable characteristics of our region’s ecology, their direct contribution to the observed surveillance patterns remains to be formally established. Consequently, while these regional climatic and demographic factors provide an interesting contextual background for our surveillance data, they should be interpreted as descriptive baseline characteristics rather than demonstrated causative drivers of the infection patterns observed in our cohort.

Ocular microbial keratitis is frequently reported to exhibit seasonal fluctuations in international literature. In our regional surveillance, the frequency of bacterial keratitis during the warmer months (52.0%) was observed to be numerically higher than in the colder seasons (48.0%); however, it is important to note that this variation did not reach statistical significance. Although the descriptive frequencies of *S. epidermidis*, *S. saprophyticus*, and *Acinetobacter spp.* appeared slightly higher during summer, and *S. pneumoniae* and *Pseudomonas spp.* showed an increased frequency in autumn, these findings do not constitute a statistically significant seasonal pattern within our study population.

Elevated environmental temperatures have been consistently linked with a rise in bacterial and fungal keratitis cases in various regions. For instance, in the Netherlands, researchers confirmed that the prevalence of acute water-associated infections such as otitis externa and the dominance of *P. aeruginosa* in freshwater lakes during summer months were associated with increased exposure to contaminated water [26]. Similarly, Khoo et al. (2020) reported a significantly higher isolation rate of *P. aeruginosa* during the dry season in Australia, although they noted that non-*Pseudomonas* bacterial keratitis did not show significant seasonal variations [27]. Furthermore, Walkden et al. (2018) investigated the correlation between season, temperature, and microbial keratitis pathogens in the UK, reporting that Gram-positive organisms in corneal scrapes were associated with warmer temperatures, while Gram-negative pathogens occurred more frequently in summer [28]. While these studies suggest significant seasonal environmental influences in their respective climates, our descriptive surveillance data highlight regional variations in pathogen isolation without establishing a definitive causal link between season and microbial prevalence.

Regarding the mechanisms of infection, genotypic analyses in broader literature have shown that *P. aeruginosa* strains isolated from bacterial keratitis can share homology with environmental strains [29]. While recreational water exposure and environmental reservoirs during hot months are recognized in other regions as factors associated with *Pseudomonas* colonization [30], our regional surveillance data primarily reflect the descriptive clinical distribution of isolates within our population rather than established direct environmental risk factors.

The selection of empirical therapy in bacterial keratitis is critical and must be guided by local susceptibility profiles. Our regional surveillance data demonstrated that *S. saprophyticus*, *Acinetobacter spp.*, and *Pseudomonas spp.* exhibited high susceptibility to ciprofloxacin, and *S. aureus* showed high susceptibility to tobramycin. Furthermore, as summarized in our surveillance records, *S. pneumoniae* isolates exhibited high susceptibility toward vancomycin (91.5%). While these descriptive susceptibility findings provide a useful baseline for local clinical considerations, they should be interpreted cautiously within the framework of regional microbiological monitoring rather than definitive resistance profiles. Ultimately, our findings highlight the value of continuous local surveillance to support empirical antimicrobial stewardship in the management of bacterial keratitis.

## Conclusion

This study provides a comprehensive **regional microbiological surveillance** and descriptive evaluation of bacterial keratitis in our cohort. Although numerical variations in isolate distributions across age groups, genders, and seasons were observed, the lack of statistical significance indicates that bacterial pathogens are persistently present across all demographic and seasonal strata. Consequently, empirical therapy and clinical management protocols should remain consistent throughout the year rather than being adjusted seasonally. While causal links with host or environmental factors could not be definitively established, our **surveillance findings** underscore the critical importance of timely microbiological evaluation, clinical vigilance, and region-specific antimicrobial selection to optimize the management of bacterial keratitis and reduce the risk of visual morbidity.

## Data Availability

No datasets were generated or analysed during the current study.
